# Soil health alterations via compost additions to natural and remediated heavy metal-contaminated mineland soils

**DOI:** 10.1007/s11356-025-36602-1

**Published:** 2025-06-03

**Authors:** Egondu C. Umeobi, Thomas F. Ducey, Mark G. Johnson, James A. Ippolito

**Affiliations:** 1 School of Environment and Natural Resources, The Ohio State University, Columbus, OH USA; 2 USDA-ARS Coastal Plain Soil, Water and Plant Conservation Research Center, Florence, SC USA; 3 US EPA Center for Public Health and Environmental Assessment, Corvallis, OR USA

**Keywords:** Heavy metal, Remediation, Compost, Soil health, Mehlich-3, 0.01 M CaCl_2_

## Abstract

The Oronogo-Duenweg mining belt in southwest Missouri is a United States Environmental Protection Agency (EPA) Superfund site due to Pb-contaminated soil and groundwater from historic mining and smelting. Remediation has removed Pb-contaminated overburden, uncovering nutrient-deficient C horizons containing elevated Cd and Zn concentrations, which impede natural revegetation. This study evaluated compost at rates of 180 and 360 Mg ha^−1^, monitoring changes in soil properties observed at naturally revegetated sites, native prairie locations (i.e., the soil health benchmark), and areas receiving compost treatments. The Soil Management Assessment Framework (SMAF) was employed to assess physical (bulk density (Bd)), water-stable aggregates (WSA)), chemical (pH, electrical conductivity (EC)), nutrient (plant-available P and K), and biological (soil organic C (SOC)), microbial biomass C (MBC), potentially mineralizable N (PMN), and β-glucosidase activity (BG)) soil health indicators and soil health scores. Soil metal concentrations were analyzed using Mehlich-3 and 0.01 M CaCl₂ extractions, while plant metals were extracted with HNO₃ and H₂O₂. Compost-treated soils exhibited overall soil health comparable to native prairie; both had greater overall soil health than the natural revegetation site. However, 360 Mg ha^−1^ rate led to excessive Mehlich-3 extractable P compared to 180 Mg ha^−1^. Mehlich-3 extractions revealed that the compost added Cd and Zn to the system, yet Cd and Zn concentrations from the 0.01 M CaCl_2_ extraction were negligible in the compost-treated soils. Plant heavy metal concentrations were below tolerable limits for livestock consumption. A target compost application rate of 180 Mg ha^−1^, or lower is suggested for balancing phosphorus and metal concentrations while improving overall soil health.

## Introduction

It is important to recognize that mining activities have a significant impact on local area ecosystem structure and function (Hu et al. 2020). Notably, mining operations can negatively affect soil organic matter/carbon content, aggregate stability, bulk density (Bd), pH, electrical conductivity (EC), plant-available nutrients, microbial activity, and bioavailable heavy metal concentrations (Sun et al. 2023; Du et al. 2023; Ippolito et al. 2024). Many, if not all these soil indicators can, in combination, provide an assessment of ecosystem functionality with respect to soil health.

Good soil health provides the necessary physical, chemical, and biological support for plant recolonization and establishment on contaminated mine tailings (Shrestha et al. 2019), and thus soil health monitoring is crucial for successfully reclaiming mining-impacted lands. However, heavy metal contaminated mine land soils can be partially or completely lacking optimal soil health attributes, including the cornerstone of soil health, soil organic carbon. Adding the right source and amount of organic matter to mining-impacted soils needs to be considered. Often, cost-effective and environmentally friendly materials can be used, such as compost.

Compost is an organic product produced through controlled aerobic, biological decomposition of organic materials (Gondek et al. 2020). Compost application is attractive because of its relatively high carbon and nutrient composition, near-neutral or slightly alkaline pH, and low bulk density, characteristics that may be completely opposite within heavy metal contaminated mine lands. Compost application to mining impacted soils has been shown to improve mine land soil physical, chemical, biological, and nutrient properties (Benidire et al. 2022; Sikdar et al. 2020; Frutos et al. 2017).

Municipal waste compost (compost sources from the organic fraction of municipal solid waste, including food scraps, yard trimmings, and other biodegradable materials collected from households, businesses, and institutions) has been used by multiple investigators. For instance, Bacchetta et al. (2015) showed that municipal waste compost (10% w/w) applied to a Cd, Pb, and Zn contaminated mine tailings waste dumps improved soil organic carbon content, cation exchange capacity, total nitrogen content, reduced total and bioavailable Cd, Pb, and Zn concentrations (by up to 9.5%), and reduced heavy metal plant uptake in *Pistacia lentiscus* and *Phragmites australis,* while improving plant survivability. Cunha-Queda et al. (2010) applied municipal waste compost (3% w/w) to a Pb and Zn contaminated mine soil, observing about a 90% reduction in extractable soil Pb and Zn. Municipal waste compost applied at 2 and 4% (w/w) to a Cd, Pb, and Zn contaminated mine soil increased *Cynara cardunculus* L. root biomass by 17- and 23-fold and shoot biomass by 5- and tenfold, respectively, while shoot Cd concentrations were reduced by 7.5-fold (Garau et al. 2021). The authors attributed increased biomass production to the compost's heavy metal sorption ability and fertility improvements (e.g., increases in plant-available P and K and soil organic C and N content).

Other types and combinations of composts have shown promise for reclaiming heavy metal-contaminated mine lands, though their effectiveness varies by source and application rate. For instance,Alvarenga et al. (2009) compared the application of municipal solid waste and garden waste composts (both at 100 or 200 Mg ha^−1^) on Cd, Pb and Zn contaminated mine land. The authors found that soil pH increased (from 3.77 in the control to 6.38 or 7.30 with 200 Mg ha^−1^ of garden waste or municipal solid waste compost, respectively) and bioavailable metal concentrations decreased with garden waste compost (from 17.9 to 12.5 and 14.8 mg kg^−1^ for Pb, and from 4.4 to 3.4 and 3.8 mg kg^−1^ for Zn with the 100 and 200 Mg ha^−1^ rates, respectively). However, the authors observed an increase in potentially bioavailable metal concentrations when municipal soil waste compost was applied (from 17.9 to 30.6 and 37.2 mg kg^−1^ for Pb, and from 4.4 to 30.3 and 40.7 mg kg^−1^ for Zn with the 100 and 200 Mg ha^−1^ rates, respectively) (Alvarenga et al. 2009). Compost made from spent mushroom compost and cattle manure compost was applied at varying rates (5, 10, 20 and 30% w/w) to two sets of mine tailings contaminated with Cd, Pb, and Zn (Sikdar et al. 2020). The authors found that heavy metal bioavailability decreased, and plant growth improved. Over both soils, compost effectively reduced Cd, Pb, and Zn concentrations by 33.1 −87.2%, 20.2–71.7%, and 6.3–80.1% compared to the control, respectively (Sikdar et al. 2020). Chiu et al. (2006) also worked with pig manure compost (2.5, 5, 10 and 20% w/w) applied to a contaminated Pb–Zn and a contaminated Cu mine tailing. The authors noted an increase in soil N, P and K concentrations, and reductions in DTPA-extractable metals in both mine tailings. The yield of *Vetiveria zizanioides* and *Phragmities australis* increased up to 10 and 5%, respectively, with compost applications (Chiu et al. 2006)*.* All the above findings are indicative of the potential benefits of amending mining impacted soils with compost. However, it is important to acknowledge potential limitations, such as the risk of introducing heavy metals through compost application (Alvarenga et al. 2009), which may impact long-term soil and environmental health. This highlights the need for a standardized framework that integrates soil health metrics to assess both the benefits and potential risks of compost use**,** thereby guiding its safe and effective application in mine land reclamation.

Frameworks have been developed to group and incorporate each soil health indicator into scoring functions to provide a comprehensive soil health assessment. Most frameworks are used for agricultural production systems, including the AgroEcosystem Performance Assessment Tool (Liebig et al. 2004), the Haney Soil Health Test (Haney et al. 2012; Yost et al. 2018), the Cornell Comprehensive Assessment of Soil Health (CASH) (Bhadha et al. 2018), and the Soil Management Assessment Framework (SMAF) (Andrews et al. 2004).

Some studies have utilized a few of these frameworks for mine land remediation. For example, Ruiz et al. (2020) used the SMAF to assess reclamation success in an open-pit limestone mine, showing improved soil health indices over a 20-year period post-reclamation. Similarly, Ippolito et al. (2024) used SMAF to evaluate long-term reclamation success of a metal-contaminated alluvial mine tailing treated with lime and biosolids. Results indicated significant improvements in soil health indicators, including increased organic carbon, plant-available nutrients, and microbial activity, along with reduced bulk density and electrical conductivity. Ippolito et al. (2024) also noted that plant metal concentrations decreased significantly, enhancing plant suitability for livestock consumption. These studies demonstrate the potential of SMAF to monitor and support sustainable reclamation efforts in metal-contaminated mine lands.

The current study aimed to evaluate reclamation success in heavy metal-affected mine soils using different compost application rates as compared to a native prairie and an untreated control soil. While compost has been widely studied for its role in metal immobilization, few studies have assessed its impact on overall soil health using an integrated and standardized framework. To address this gap, we applied the Soil Management Assessment Framework (SMAF) to evaluate physical, chemical, nutrient, and biological indicators of soil health. By combining these assessments with measures of soil heavy metal availability, this study offers a more comprehensive evaluation of compost-based reclamation strategies in contaminated mine soils.

## Methods

**Fig. 1:**
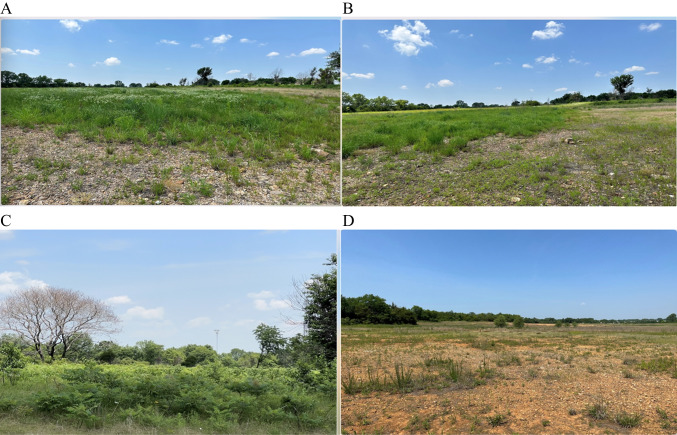
The following are the designated sites used for the compost remediation study. **A**) 360 Mg compost ha^−1^, **B**) 180 Mg compost ha^−^^1^, **C**) Native Prairie, **D**) Natural  Revegetation

The study location is near Webb City in Jasper County, Missouri, and is part of the Oronogo-Duenweg mining belt in southwest Missouri, United States of America (USA). The study area was designated by United States Environmental Protection Agency as the Oronogo-Duenweg Superfund site in 1990. The area sits at approximately 300 m above sea level, receives an annual average precipitation of 1,143 mm, and experiences temperatures ranging from −6 °C to 32 °C throughout the year. Like many locations in the area, the study site was covered with old mine tailings and smelter wastes from over one hundred years of Pb mining and smelting operations. The study site underwent initial remediation by removing Pb-contaminated overburden to a point where the Pb concentration in the residual soil, or subsoil, was < 400 mg kg^−1^ (as determined using a hand-held x-ray fluorescence spectrometer). To achieve this acceptable level of Pb often required the removal of contaminated O, A, and most of the B horizons leaving C horizons at the soil surface. Unfortunately, these subsoils typically contained excessive Cd and Zn concentrations, likely due to years of leaching from the overburden. In combination with a lack of organic matter, nutrients, microbial activity, heavy clay content, and pebble/cobble content, these subsoils are recalcitrant to revegetation efforts (Fig. 1).

In 2016, two ~ 0.2 ha sites were treated with lime at a rate of 13.9 Mg ha^−1^, along with compost (a blend of cattle manure, green waste, vegetable waste, and wood chips) applied at rates of 180 or 360 Mg ha^−1^. Both lime and compost were surface applied with no incorporation as incorporation is nearly impossible given site conditions (e.g., high percentage of coarse fragments and heavy texture) at the soil surface. Notably, the compost contained 15.8 mg Cd kg^−1^, 73 mg Pb kg^−1^, and 7,085 mg Zn kg^−1^, with elevated concentrations a function of locally sourced compost feedstock materials. After compost application, the compost plots were reseeded with a tallgrass prairie mixture.

The sites were visited in November 2022 for soil and plant sampling. In addition to the two compost sites, two additional on-site locations were chosen to represent: 1) post soil removal but allowed to naturally revegetate itself (referred to as Natural Revegetation; 0 Mg ha^−1^ compost); and 2) a Native Prairie (no past disturbance or soil removal, and no compost or lime application) with the latter a target benchmark for soil health reclamation success. As replicating the Natural Revegetation and Native Prairie locations was impossible at the location, and the 180 and 360 Mg ha^−1^ compost applications were applied in only two areas, a pseudo-replication sampling design was used to collect soil and plant samples from within all four locations. Due to the pseudo-replicated design, results reflect within-site variability but may not fully capture broader spatial variation. Areas within all four locations encompassed a total ~ 0.2 ha, and these areas were split into fourths with soil and plant sampling occurring within each sub-area (i.e., four pseudo-replicates per treatment; 16 samples total were collected).

At each sampling location, soil samples for bulk density (Bd) and gravimetric moisture content were collected using a corer that collected undisturbed soil samples in 5.7 cm diameter and 3.0 cm tall brass rings. Samples were placed in drying tins, weighed immediately, then oven-dried at 105 °C for 24 h and weighed again. At each sampling point, soil from the 0–15 cm depth was collected using a shovel, stored in Ziploc bags, placed in coolers with ice packs, and immediately returned to the lab. In the lab, all soils were passed through an 8-mm sieve, and ~ 150 g of field-moist soil was stored at 4 °C. Approximately 300 g of the 8-mm sieved soil was passed through a 2-mm sieve and allowed to air dry. The remaining 8-mm sieved soil was also allowed to air dry. Once air-dried, about 20 g of the 2-mm sieved soil was powder-ground. The SMAF was used to evaluate soil health by condensing various soil indicators into a minimal dataset. This framework, described in detail by Andrews et al. (2004) and further explored in studies by Buchanan and Ippolito (2021) and Trimarco et al. (2023), assesses key soil characteristics such as Bd, wet aggregate stability (WSA), pH, electrical conductivity (EC), plant-available nutrients (for this site, Mehlich-3 extractable P and K), potentially mineralizable nitrogen (PMN), microbial biomass carbon (MBC), β-glucosidase activity (BG), soil organic carbon (SOC), and clay content. Detailed analyses for each indicator are presented in Ippolito et al. (2024). Following indicator analyses, indicators are entered into SMAF and are scored from 0.00 (i.e., “worst”) to 1.00 (i.e., “best”) using a”more is better”, “less is better”, or “optimal range” is better approach. These scores are then weighted to determine a soil physical, chemical, nutrient, biological, and overall soil health score ranging from 0.00 to 1.00.

Soil heavy metal bioavailability was determined by placing 3.00 g of 2-mm sieved, air-dried soil into a 50 mL centrifuge tube, adding 30 mL of 0.01 M CaCl₂, and shaking at 120 rpm for 2 h (Ippolito et al. 2014). The mixture was centrifuged at 500 × *g* and the supernatant filtered through a 0.45 μm membrane, and analyzed for Cd, Pb, and Zn using inductively coupled plasma-optical emission spectroscopy (ICP-OES). Plant-available metals were assessed using a Mehlich-3 extraction (Mehlich 1984), with extracts analyzed by ICP-OES.

Since 2016, the natural revegetation site has been dominated mostly by *Ambrosia artemisiifolia* (annual ragweed), while the compost sites and the native prairie have been dominated by *Andropogon gerardii* (big bluestem), *Apocynum cannabinum* (dogbane), *Helianthus maximiliani* (Maximilian sunflower), *Rudbeckia subtomentosa* (sweet coneflower), *Sorghastrum nutans* (Indian grass), *Panicum virgatum* (switchgrass), *Galium aparine* (bedstraw), *Ratibida pinnata* (grey-headed coneflower), *Solidago altissima* (tall goldenrod), *Cynodon dactylon* (Bermuda grass), *Coronilla varia* (crown vetch), *Barbarea vulgaris* (yellow rocket), and *Conyza canadensis* (horseweed). Aboveground plant biomass was collected using a 0.5 m2 quadrat at a 5 cm height, dried at 60 °C for 78 h, weighed, ground, and digested with concentrated HNO₃ and 30% H₂O₂ (Huang and Schulte 1985). The digestate was then analyzed for Cd, Pb, and Zn using ICP-OES.

### Statistical analysis

Data analysis was conducted using R Studio (version 4.2.0). Four pseudoreplicate samples were used for each site, with each site representing a treatment. All data underwent normality and homoscedasticity tests, with transformations applied as needed. Square root transformations were used for BG, C, EC, K (Mehlich-3), Bd, and Cd (Mehlich-3 and 0.01 M CaCl₂ extractable). To normalize the data log transformations were applied to Cd and Pb plant metal uptake, P and Pb (Mehlich-3 extractable), Zn (0.01 M CaCl₂ extractable), and plant Cd concentration. ANOVA was performed at a significance level of p < 0.05, followed by Tukey-adjusted pairwise comparisons to identify differences among the two composted, naturally revegetated, and native prairie sites. Data presented in tables and figures are untransformed.

Pearson correlation analysis was also used to evaluate relationships between plant total, Mehlich-3 extractable, and 0.01 M CaCl_2_ bioavailable metal concentrations. Significance was determined at p-values of 0.05, 0.01, and 0.001. The bioaccumulation coefficient (BAC), a measure that suggests how metals accumulate in plants as compared to soils, was calculated as the ratio of plant Cd, Pb, and Zn concentrations to the Mehlich-3 extractable soil Cd, Pb, and Zn concentrations (Ali et al. 2019; Umeobi et al. 2024).

1 $$\text{BAC }= {~}^{Metal\;concentration\;in\;the\;plant}\!\left/ \!{~}_{Metal\;concentration\;in\;the\;soil}\right.$$

## Results and discussion

### Soil health indicators and indicator scores

The Soil Management Assessment Framework (SMAF) utilizes soil health indicators as a first step in quantifying soil health, with indicators converted into unitless scores ranging from 0.00 (“worst”) to 1.00 (“best”). Table 1 presents all SMAF soil health indicator data and individual indicator scores, showing significant differences across sites for WSA, Bd, pH, EC, PMN, SOC, MBC, P, and K. The majority of these differences suggest that compost application contributed to changes in soil health, aiding reclamation success. Recent studies (Ippolito et al. 2024; Mukhopadhyay et al. 2014, 2016) have identified similar key indicators of reclamation success, with variations influenced by local soil and tailings characteristics, and site-specific factors. Specific differences in physical, chemical, biological, and nutrient indicators and scores are discussed below.

- 1) Physical soil health indicators and scores

**Table 1:** Mean Soil Management Assessment Framework (SMAF) physical [bulk density (Bd) and wet aggregate stability (WAS)], chemical [pH and electrical conductivity (EC)], nutrient [Mehlich-3 extractable P and K (M3P and M3 K)], and biological [soil organic C (SOC), potentially mineralizable N (PMN), microbial biomass C (MBC), and β-glucosidase activity(BG)] indicators and SMAF indicator scores as a function of mine tailing reclamation location. Values inside parenthesis represent the standard error of the mean (*n* = 4). Within a column, different lowercase letters after an individual indicator or a SMAF indicator score indicate a significant difference as determined by a Tukey adjusted pairwise comparison. N/A = not applicable due to 0.00 standard error of the mean

Sites Location	Physical indicator		Chemical indicators		Biological Indicators				Nutrients indicators	
	WSA *(%)*	Bd *(g cm*^*−3*^*)*	pH	EC *(dS m*^*−1*^*)*	ΒG *(ug g*^*−1*^* h*^*−1*^*)*	PMN *(mg kg*^*−1*^*)*	SOC *(%)*	MBC *(m/kg*^*−1*^*)*	M3P *(mg kg*^*−1*^*)*	M3 K *(mg kg*^*−1*^*)*
360 Mg ha^−1^ Compost	64.2(5.98)^a^	0.705(0.03)^a^	6.82(0.05)^b^	0.58(0.10)^b^	472(279)^a^	43(8)^b^	7.18(1.54)^b^	371(26)^b^	355(110)^c^	359(65)^b^
180 Mg ha^−1^ Compost	74.5(4.42)^a^	0.782(0.08)^ab^	7.12(0.06)^b^	0.32(0.02)^b^	130(28)^a^	14(10)^a^	3.84(0.99)^ab^	298(61)^b^	92(41)^b^	199(26)^a^
Native Prairie	91.2(0.85)^b^	0.912(0.04)^ab^	5.77(0.11)^a^	0.05(0.02)^a^	767(242)^a^	57(2)^b^	4.82(0.47)^b^	486(59)^b^	20(3)^b^	132(8)^a^
Natural Revegetation	78.5(1.89)^ab^	0.993(0.09)^b^	5.42(0.25)^a^	0.07(0.02)^a^	879(600)^a^	3(3)^a^	0.76(0.17)^a^	51(15)^a^	1(0)^a^	180(21)^a^
*ANOVA* *P-value*	*0.003*	*0.032*	< *0.001*	< *0.001*	*0.478*	< *0.001*	*0.004*	< *0.001*	*0.004*	*0.003*
SMAF Indicator Scores (scale of 0.00–1.00)										
360 Mg ha^−1^ Compost	1.00(0.00)^a^	0.99(0.00)^a^	0.95(0.01)^b^	0.99(0.01)^a^	0.53(0.27)^a^	1.00(0.00)^b^	1.00(0.0)^b^	0.92(0.03)^c^	0.36(0.22)^a^	0.99(0.01)^b^
180 Mg ha^−1^ Compost	1.00(0.00)^a^	0.99(0.00)^a^	0.82(0.03)^ab^	1.00(0.00)^a^	0.37(0.15)^a^	0.33(0.23)^a^	0.81(0.11)^b^	0.52(0.18)^b^	0.93(0.07)^b^	0.95(0.02)^ab^
Native Prairie	1.00(0.00)^a^	0.99(0.00)^a^	0.76(0.06)^ab^	1.00(0.00)^a^	0.81(0.19)^a^	1.00(0.00)^b^	0.99(0.00)^b^	0.95(0.03)^c^	0.91(0.05)^b^	0.89(0.02)^a^
Natural Revegetation	1.00(0.00)^a^	0.90(0.07)^a^	0.57(0.14)^a^	1.00(0.00)^a^	0.64(0.21)^a^	0.22(0.21)^a^	0.08(0.02)^a^	0.05(0.01)^a^	0.00(0.00)^a^	0.96(0.021)^ab^
*ANOVA* *P-value*	*NA*	*0.158*	*0.029*	*0.426*	*0.522*	*0.004*	< *0.001*	< *0.001*	< *0.001*	*0.034*

Compost application significantly improved physical soil health indicators. Bulk density decreased by approximately 30% across sites treated with compost (180 and 360 Mg ha^−1^) compared to untreated sites, potentially contributing to enhanced permeability. These improvements align with Cai et al. (2021), who reported that incorporating 5% (w/w) compost and CaCO_3_ reduced Bd and increased soil permeability, with the compost application enhancing the abundance of cation exchange sites, thereby increasing heavy metal adsorption; this may have been occurring in the current study. There were no differences between the Bd indicator scores. It also appears that compost has yet to effect WSA, as there were no significant differences in WSA indicator scores across sites. As a reference, any WSA value above 51% is interpreted as optimal within SMAF (Table 1).

- 2) Chemical soil health indicators and scores

Soil pH increased by 1 to 2 units with compost application as compared to the native prairie and natural revegetation sites, creating a more favorable environment for microbiological activity and plant growth as discussed below (Table 1). These pH differences led to differences in the pH indicator scores, with the highest compost application rate leading to a greater score as compared to the natural revegetation site. Compost-treated sites also showed slight increases in EC as compared to the native prairie and natural revegetation sites. However, these EC values are well below those considered an issue for most plant species (Carter and Gregorich 2008), as reflected in all EC indicator scores being similar across sites.

- 3) Biological soil health indicators and scores

Biological indicators demonstrated substantial improvements in compost-treated soils, including enhanced PMN, SOC, and MBC; no differences existed for BG activity across sites (Table 1). It is interesting to note that BG activity was unexpectedly elevated in the naturally revegetated site. This may have been due to the preference of BG activity falling within a pH range of 4.5 to 6.5 (Kok et al. 2012), or microbial communities in the natural revegetation site being better adapted to local conditions. The overall increase in PMN, SOC, and MBC highlights improved microbial activity, critical for organic matter decomposition, turnover and nutrient. Furthermore, the differences reflected in the indicators are similarly reflected in the indicator scores, suggesting that SMAF is sensitive to these biological soil health indicators across these sites.

- 4) Nutrient soil health indicators and scores

The nutrient indicators, P and K, significantly increased with increasing compost application rates. Sites treated with 360 Mg ha^−1^ of compost outperformed those treated with 180 Mg ha^−1^, with P and K concentrations levels exceeding those of native prairie and natural revegetation sites (Table 1). These findings align with prior studies (Garau et al. 2021; Ippolito et al. 2024) that demonstrated increased P and K concentrations following compost or organic amendment applications to mine tailings. Although the K indicator scores followed those of the other indicators, the P indicator scores showed that the natural revegetation site had the lowest score due to a severe lack of plant-available P. The highest compost application rate also showed a significant decline in the P indicator score, driven by excessive plant-available P concentrations that could potentially lead to environmental concerns. Thus, a lower compost application rate (e.g., 180 Mg ha^−1^ or lower) may be, overall, more beneficial from a plant-available P standpoint and reducing the potential for the runoff or leaching of excess P.

- 5) Physical, chemical, biological, nutrient, and overall soil health scores

**Fig. 2:**
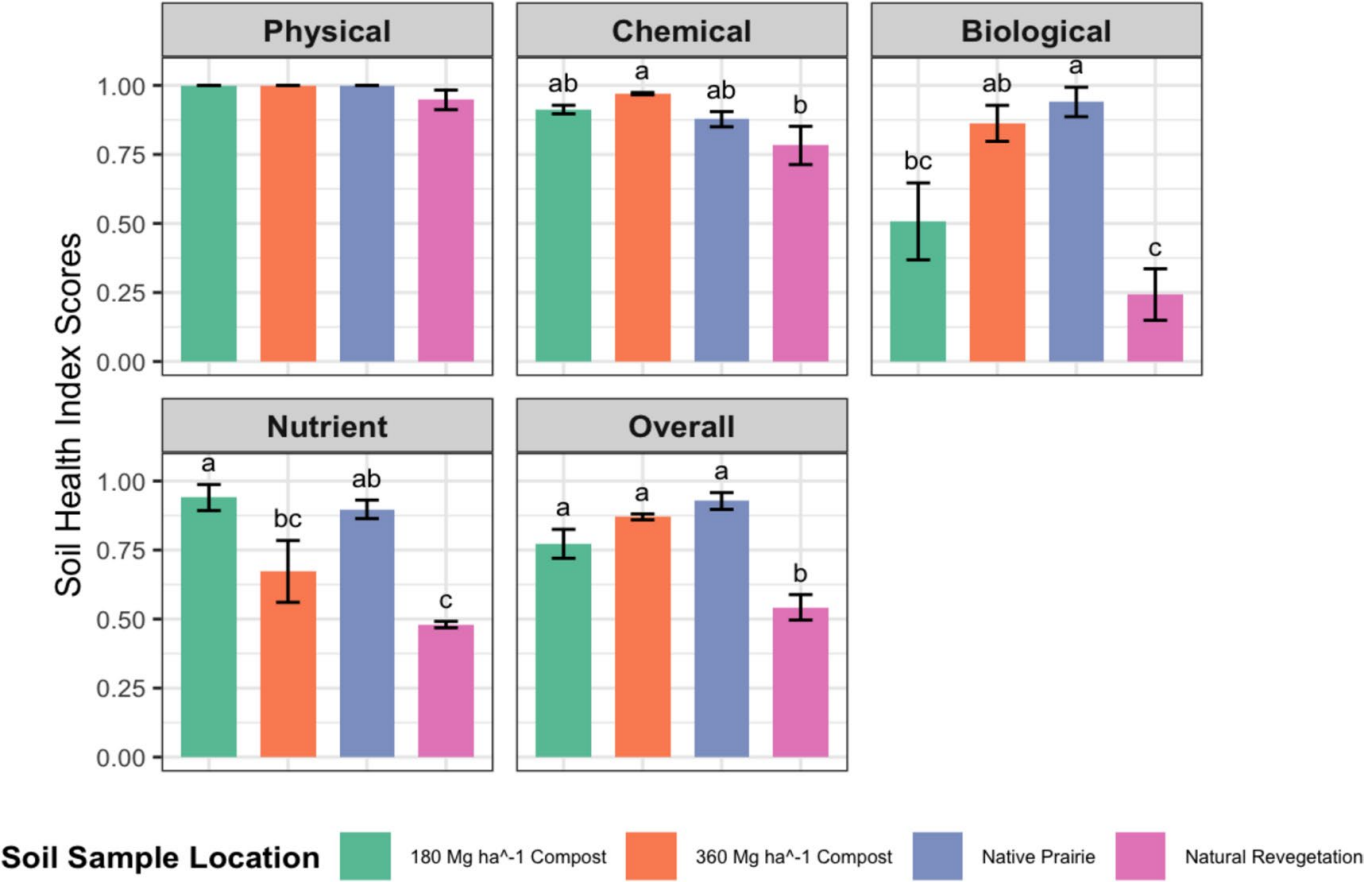
Soil physical, chemical, nutrient, biological, and overall soil health indices as a function of applying 360 or 180 Mg compost ha^−1^, or within native prairie or natural revegetation sites. Bars represent soil health index scores for different soil sample locations across the five categories. Error bars represent the standard error of the mean. Different letters above bars indicate significant differences (p < 0.05) as determined by a Tukey adjusted pairwise comparison

Compost application at rates of 180 and 360 Mg ha^−1^ did not affect soil physical health, but significantly enhanced chemical, biological, nutrient, and overall SMAF soil health scores (Fig. 2). Details regarding each soil health score are provided below.

A) Physical Soil Health Scores

The physical soil health scores, influenced by Bd and WSA, showed no significant differences across the study sites (Fig. 2). Although Bd decreased with increasing compost application as compared to the natural revegetation site, the WSA was maximized over all sites, signifying relatively high aggregate stability regardless of treatment. This result underscores the resilience of soil structure across the experimental conditions, potentially masking any physical soil health differentiation expected with compost applications.

- B) Chemical soil health scores

The chemical soil health scores, influenced by pH and EC, showed that compost application led to notable improvements in the chemical soil health score. This difference was driven primarily by increases in soil pH and not EC, as all EC indicators scores were similar across sites (Fig. 2). Soil pH increased by over 2 units, neutralizing the acidity of the contaminated mine tailings and likely creating a more favorable environment for plant growth and microbial activity.

- C) Biological soil health scores

The biological soil health scores, influenced by BG, PMN, SOC, and MBC, showed that the highest compost application rate was equal to the native prairie (i.e., the soil health benchmark); the 180 Mg ha^−1^ compost application was similar to the highest compost application and the natural revegetation site (Fig. 2). The outcome following weighting the biological soil health indicators points towards enhanced microbial activity, nutrient cycling and turnover, all critical for long-term reclamation success. A similar outcome was suggested by Ippolito et al. (2024) when using SMAF for monitoring long-term reclamation success of mine tailings.

- D) Nutrient soil health scores

The nutrient soil health scores, influenced by plant-available P and K concentrations, improved with compost application as compared to the natural revegetation site (Fig. 2). Moreover, the 180 Mg ha^−1^ compost application rate had a similar nutrient soil health score as compared to the native prairie site (i.e., the soil health benchmark). The 360 Mg ha^−1^ compost application rate was also similar to the native prairie but was lower than the 180 Mg ha^−1^ compost application rate due to excessive plant-available soil P concentrations. In fact, P concentrations in the highest compost site exceeded the maximum P concentrations set forth in SMAF (~ 300 mg kg^−1^), considered as extremely excessive and potentially leading to environmental degradation. This one finding alone suggests that while compost rates may boost nutrient availability, they may also lead to nutrient imbalances, necessitating careful application rate considerations for future reclamation purposes.

- E) Overall soil health scores

The overall soil health scores, a weighted average of all physical, chemical, biological, and nutrient indicator scores, reflects the cumulative soil health improvements across sites (Fig. 2). Both compost-treated sites demonstrated overall soil health equal to the native prairie (i.e., the soil health benchmark) and improved overall soil health compared to the natural revegetation site. The natural revegetation site lagged behind the other three sites due to limited improvements in chemical, biological, and nutrient soil health. These findings highlight the importance of targeted interventions like compost application for effective mine land reclamation success. Among the compost-treated sites and based on above soil health findings, the 180 Mg ha^−1^ rate provided an optimal balance across soil health indicators, demonstrating successful reclamation five years since application. At the very least, 180 Mg ha^−1^ compost application rates could be used at similar sites within the Oronogo-Duenweg mining belt, although even lower compost application rates should be studied to potentially reclaim more areas within the region. With lower compost application rates more acres of remediated soils can be treated with compost across the region, thereby reducing the area of barren, remediated and unamended soils.

### Plant biomass, Cd, Pb, and Zn concentrations and uptake, and bioaccumulation coefficient (BAC)

Plant biomass, Cd, Pb, and Zn concentrations and uptake, and the BAC are presented in Table 2. Biomass production across the sites varied; the 180 Mg compost ha^−1^ and the native prairie sites produced the greatest biomass, biomass from the 360 Mg compost ha^−1^ site was similar to the native prairie yet lower than the 180 Mg compost ha^−1^, and the natural revegetation site produced the least biomass. The higher biomass at the 180 Mg ha⁻1 site likely reflected optimal nutrient and soil conditions, while the slightly lower biomass at 360 Mg ha⁻1 may have been a result of nutrient imbalances. The natural revegetation site had the lowest biomass, reflecting poor soil health conditions. Similar observations have been made by many others (e.g., Garau et al. 2021; Brown et al. 2009; Fisher et al. 2000). Plant tissue metal concentrations, however, were similar across all sites. Even though plant tissues contained detectable concentrations of Cd and Zn, and to a much lesser degree Pb, all metal concentrations were below the National Research Council (2005) maximum tolerable levels in animal feed (10, 10, and 250 mg kg^−1^ minimum Cd, Pb, and Zn concentrations, respectively). Plant metal uptake followed the general order of 360 Mg compost ha^−1^ > 180 Mg compost ha^−1^ > native prairie > natural revegetation, suggesting that the greater the biomass the greater metal uptake (Table 2). Although not investigated in this study, these values could represent greater root penetration into the underlying soil. On the one hand, plant metal uptake is increased. On the other, roots penetrating into the subsoils adds organic matter that leads to reduced Zn and Cd bioaccessibility and increased soil stabilization and subsequent reduction in erosion and promotion of overall system recovery. The bioaccumulation coefficient (BAC), which represents the ratio of metal concentration in plants to Mehlich-3 soil extractable metal concentration, indicate that BAC values were similar across all four sites. The BAC for Cd, Pb, and Zn ranged from 0.00–0.85, 0.00–0.19, and 0.32–16.96, respectively, across all study sites (Table 2). It is interesting to note that composts utilized for reclamation purposes contained elevated Cd and Zn concentrations, yet the BAC results suggest that metal bioaccumulation was not affected. This suggests that metals present in compost were not readily bioavailable, supporting the use of the 0.01 M CaCl_2_ soil extraction to quantify soil metal bioavailability in the context of soil health for mine land reclamation success. Overall, greater above-ground biomass, concomitantly with no significant increase in plant metal concentration or bioaccumulation, should lead to greater ground cover and reduce offsite sediment and metal transport. These improvements should reduce health-related risks for plants, animals, and humans, demonstrating the potential of compost as a sustainable amendment for mine land reclamation (Chiu et al. 2006; Bacchetta et al. 2015).

**Table 2:** Plant biomass, metal concentrations, metal uptake, and metal bioaccumulation coefficient. All values inside parenthesis represent the standard error of the mean (*n* = 4). Different lowercase letters after an individual heavy metal concentration indicate a significant difference as determined by a Tukey-adjusted pairwise comparison

Sites Location	Biomass	Plant Metal Concentration Cd Pb Zn			Plant Metal Uptake Cd Pb Zn			Metal Bioaccumulation Coefficient(BAC) Cd Pb Zn		
	*kg ha*^−1^	–––––––- *mg kg*^-1^ –––––––-			–––––––- *g ha*^−1^ –––––––-					
360 Mg ha^−1^ Compost	*1446(105)*^*b*^	4.06(3.97)^a^	0.00(0.00)^a^	77.9(25.7)^a^	9.70(9.40)^a^	0.00(0.00)^a^	105.8(26.9)^ab^	0.40(0.39)^a^	0.00(0.00)^a^	0.42(0.19)^a^
180 Mg ha^−1^ Compost	*1894(47)*^*a*^	0.64(0.53)^a^	0.00(0.00)^a^	73.8(14.1)^a^	2.20(1.67)^a^	0.00(0.00)^a^	138(23.5)^a^	0.25(0.21)^a^	0.00(0.00)^a^	0.32(0.08)^a^
Native Prairie	*1635(32)*^*ab*^	0.08(0.08)^a^	0.02(0.02)^a^	60.6(16.6)^a^	0.53(0.00)^a^	0.09(0.00)^a^	99.7(27.7)^ab^	0.00(0.00)^a^	0.00(0.00)^a^	0.59(0.18)^a^
Natural Revegetation	*748(167)*^*c*^	0.60(0.25)^a^	0.55(0.40)^a^	47.7(9.8)^a^	0.40(0.18)^a^	0.40(0.29)^a^	31.7(2.6)^b^	0.85(0.34)^a^	0.19(0.16)^a^	17.0(13.7)^a^
*ANOVA* *P-value*	< *0.001*	*0.261*	*0.206*	*0.622*	*0.478*	*0.672*	*0.037*	*0.240*	*0.292*	*0.278*

### Soil Mehlich-3 and 0.01 M CaCl_2_ extractable heavy metals, and correlations to plant metal concentrations

**Fig. 3:**
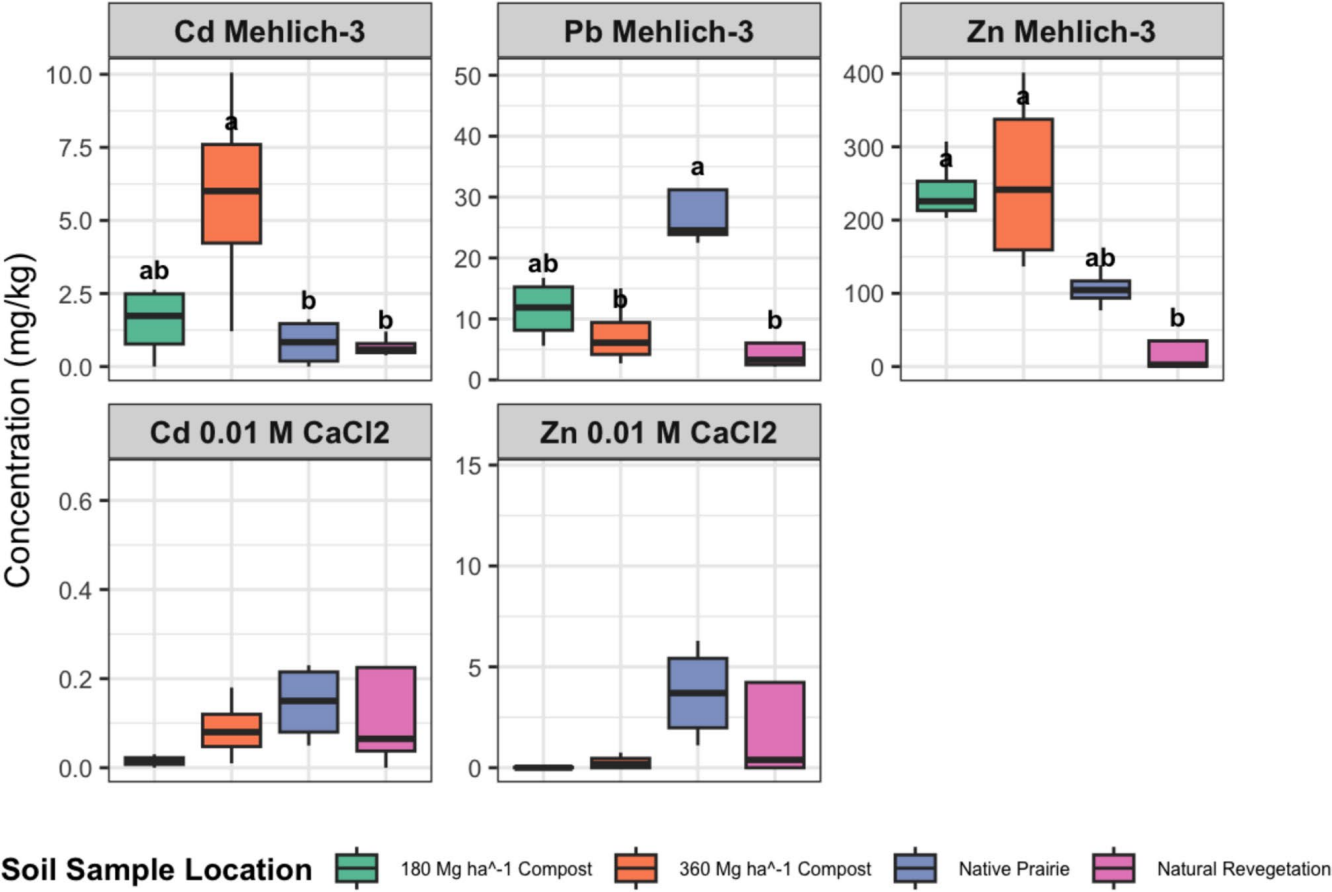
Mehlich-3 extractable soil Cd, Pb, and Zn concentrations, and 0.01 M CaCl_2_ extractable Cd and Zn concentrations (0.01 M CaCl_2_ extractable Pb concentrations were below detection; data not shown), as a function of applying 360 or 180 Mg compost ha^−1^, or within native prairie or natural revegetation sites. Box plots represent the interquartile range with the bold line indicating the median. Different letters above bars indicate significant differences (p < 0.05) as determined by a Tukey adjusted pairwise comparison

Figure 3 illustrates the Mehlich-3 extractable and 0.01 M CaCl₂ extractable Cd, Pb, and Zn concentrations. Compost applications significantly increased Mehlich-3 extractable Cd and Zn, likely a function of locally sourced feedstock materials used for compost creation containing elevated Cd and Zn concentrations. Similar increases in Zn concentrations following compost application have been reported by Alvarenga et al. (2009) and Spargo and Doley (2016), particularly when compost was sourced from municipal solid waste. The concentrations of both metals in compost-treated sites exceeded the world average soil value for Cd (0.41 mg kg^−1^) and Zn (70 mg kg^−1^) (Kabata-Pendias 2011). However, the Zn concentration remained below the 300 mg kg^−1^ threshold for agricultural soils as reported by Kabata-Pendias (2011). The Mehlich-3 extractable Pb concentrations in compost-treated sites were below the world average soil value of 27 mg kg^−1^ (Kabata-Pendias 2011), a reflection of Pb-contaminated O, A, and B horizon soil removal during remediation. It is interesting to note that the elevated soil Pb concentration in the native prairie may have resulted from air deposition (i.e., dust) during nearby mining activities or during mine site remediation.

**Fig. 4:**
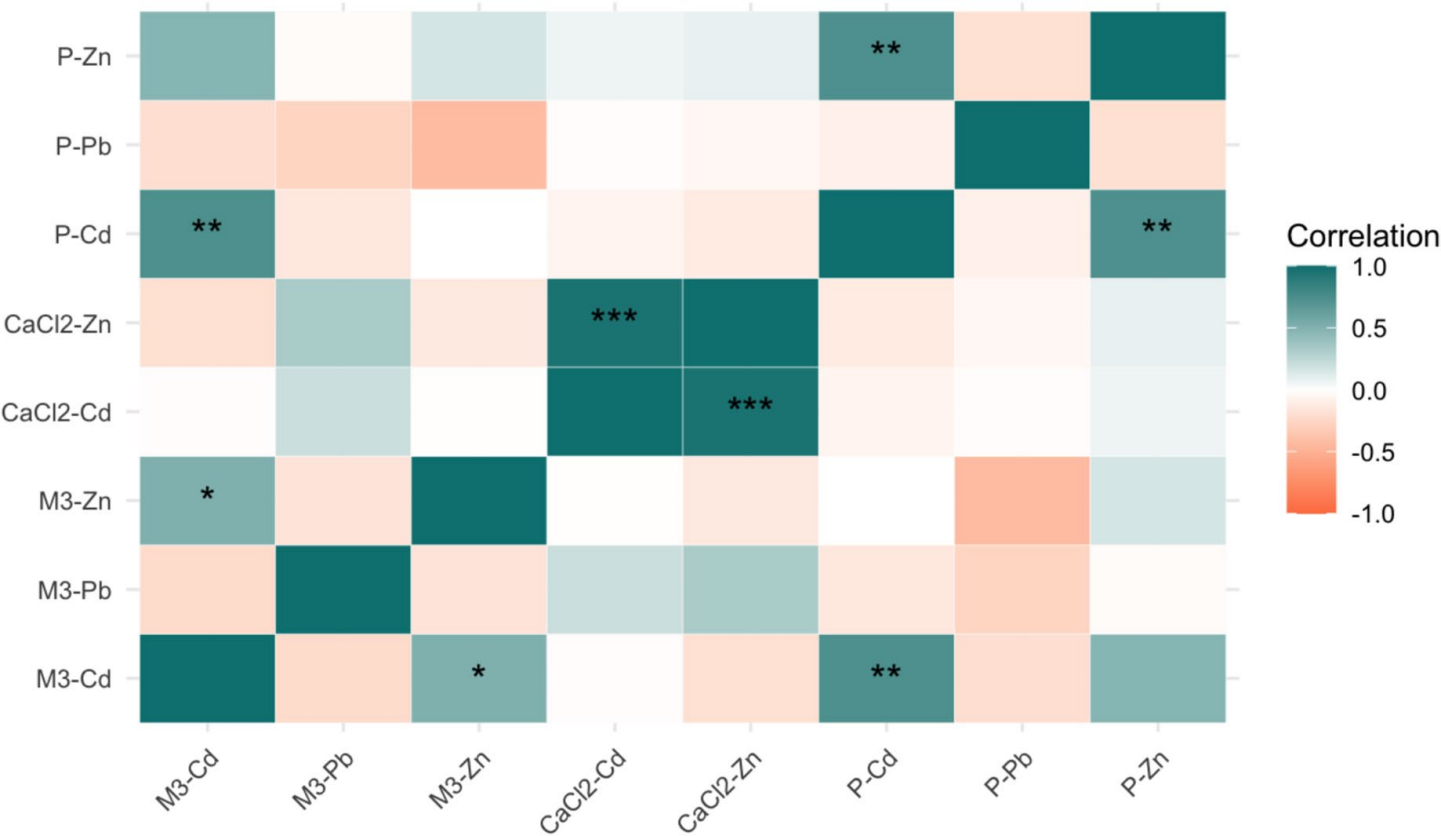
Correlations between plant and soil metal concentration (*P-value* < 0.001,"***", *P-value* < 0.01,"**", *P-value* < 0.05,"*"). Note: M3-Cd (Mehlich-3 extractable Cd), M3-Pb (Mehlich-3 extractable Pb), M3-Zn (Mehlich-3 extractable Zn), CaCl2-Cd (0.01 M CaCl_2_ extractable Cd), CaCl2-Pb (0.01 M CaCl_2_ extractable Pb), CaCl2-Zn (0.01 M CaCl_2_ extractable Zn), P-Cd(plant-Cd), P-Pb (plant-Pb) and P-Zn(plant-Zn)

The 0.01 M CaCl₂ extractable Cd and Zn concentrations showed no significant differences between sites, and the 0.01 M CaCl₂ extractable Pb concentrations were below the detection limit (data not shown). Thus, the 0.01 M CaCl₂ extraction may not always capture the full extent of plant-available or environmentally mobile metal fractions in (reclaimed) metal-contaminated soils (Fig. 3). Despite this, correlation analysis between plant metal concentrations (Cd, Pb, Zn) and soil extractions (Fig. 4) indicated that plant Cd concentrations were correlated with Mehlich-3-extractable Cd, while no significant correlations were observed for plant Zn or Pb with either Mehlich-3 or 0.01 M CaCl₂ extractable fractions. This suggests that these results are mixed. Consequently, future studies could integrate both extraction methods to provide complementary insights for using Mehlich-3 to assess broader soil nutrient availability while using 0.01 M CaCl₂ for assessing metal bioavailability in soil health evaluations.

## Conclusions and recommendations

Historic mining operations often lead to heavy metal contamination in the environment, posing risks to soil, plant, animal, and human health. Within the Oronogo-Duenweg mining belt in southwest Missouri, USA, compost applications have emerged as an effective remediation strategy for restoring soil health. The application of compost at rates of 180 and 360 Mg ha^−1^ successfully restored overall soil health (i.e., a combination of soil physical, chemical, biological, and nutrient soil health) to levels comparable to a local native prairie (i.e., the soil health benchmark), indicating effective reclamation. No differences in soil bioavailable Cd, Pb, or Zn concentrations were observed across sites when extracted with 0.01 M CaCl_2_. However, a Mehlich-3 extraction revealed variations among compost-treated, native prairie, and natural revegetation sites, suggesting that Mehlich-3 is too harsh of an extractant for this particular location. Plant Cd, Pb, and Zn concentrations, and BAC, showed no differences between sites, suggesting that if a soil extraction needs to be selected to identify potential plant metal uptake, the 0.01 M CaCl_2_ extraction is a better choice (as compared to Mehlich-3) in this area. Importantly, plant Cd, Pb, and Zn concentrations were below maximum tolerable limits for animals, demonstrating the safety and efficacy of compost treatments for soil remediation. Based on all findings, the 180 Mg ha^−1^ of compost appears to be a most suitable choice for heavy metal contaminated mine land soil reclamation in this area. However, even lower compost application rates might still achieve soil health improvements, comparable to the native prairie, while maximizing environmental benefits. Lower compost application rates should be studied as they would extend compost utilization across larger areas, particularly in expansive regions like the 6,500 square km Oronogo-Duenweg mining belt.

## Data Availability

All data will be available on request.

## References

[CR1] Ali H, Khan E, Ilahi I (2019) Environmental chemistry and ecotoxicology of hazardous heavy metals: environmental persistence, toxicity, and bioaccumulation. J. Chem. 2019:6730305 (Carter and Gregorich, 2008)

[CR2] Alvarenga P, Gonçalves AP, Fernandes RM, de Varennes A, Duarte E, Cunha-Queda AC, Vallini G (2009) Reclamation of a mine contaminated soil using biologically reactive organic matrices. Waste Manag Res 27:101–111. 10.1177/0734242X08091556 19244409 10.1177/0734242X08091556

[CR3] Andrews SS, Karlen DL, Cambardella CA (2004) The soil management assessment framework: a quantitative soil quality evaluation method. Soil Sci Soc Am J 68:1945–1962

[CR4] Bacchetta G, Cappai G, Carucci A, Tamburini E (2015) Use of native plants for the remediation of abandoned mine sites in mediterranean semiarid environments. Bull Environ Contam Toxicol 94:326–333. 10.1007/s00128-015-1467-y 25626521 10.1007/s00128-015-1467-y

[CR5] Benidire L, Pereira S, Aboudrar W, Hafidi M, Castro P, Boularbah A (2022) Remediation of metal-contaminated mine tailings by the application of organic and mineral amendments. J Soils Sediments 22:482–495. 10.1007/s11368-021-03081-z

[CR6] Bhadha J, Khatiwada R, Galindo S, Xu N, Capasso J (2018) Evidence of soil health benefits of flooded rice compared to fallow practice. Sustain Agric Res 7:31. 10.5539/sar.v7n4p31

[CR7] Brown S, Svendsen A, Henry C (2009) Restoration of high zinc and lead tailings with municipal biosolids and lime: a field study. J Environ Qual 38:2189–2197. 10.2134/jeq2008.0103 19875774 10.2134/jeq2008.0103

[CR8] Buchanan CM, Ippolito JA (2021) Long-term biosolids applications to overgrazed rangelands improve soil health. Agronomy 11:1339. 10.3390/agronomy11071339

[CR9] Cai B, Chen Y, Du L, Liu Z, He L (2021) Spent mushroom compost and calcium carbonate modification enhances phytoremediation potential of Macleaya cordata to lead-zinc mine tailings. J Environ Manag 294:113029 10.1016/j.jenvman.2021.113029 34126537

[CR10] Carter MR, Gregorich EG (2008) Soil sampling and methods of analysis, 2nd edn. CRC Press, Boca Raton (Fla)

[CR11] Chiu K, Ye Z, Wong M (2006) Growth of vetiveria zizanioides and phragmities australis on Pb/Zn and Cu mine tailings amended with manure compost and sewage sludge: a greenhouse study. Bioresour Technol 97:158–170. 10.1016/j.biortech.2005.01.038 16154513 10.1016/j.biortech.2005.01.038

[CR12] Cunha-Queda C, Alvarenga P, Nobre A, de Varennes A (2010) Effect of Municipal solid waste compost on mine soils as evaluated by chemical, biological and biochemical properties of soil. Compost Sci Util 18:89–96. 10.1080/1065657X.2010.10736940

[CR13] Du B, Zhang H, Ji D, Huang Z, Fangqun G, Zhou J (2023) Environmental contamination and health risk assessment to toxic elements in an active lead-zinc mining area. Expo Health 15:687–698. 10.1007/s12403-022-00515-y

[CR14] Fisher KT, Brummer JE, Leininger WC, Heil DM (2000) Interactive effects of soil amendments and depth of incorporation on Geyer willow. J Environ Qual 29:1786

[CR15] Frutos I, García-Delgado C, Cala V, Gárate A, Eymar E (2017) The use of spent mushroom compost to enhance the ability of Atriplex halimus to phytoremediate contaminated mine soils. Environ Technol 38:1075–1084. 10.1080/09593330.2016.1217938 27494563 10.1080/09593330.2016.1217938

[CR16] Garau M, Castaldi P, Patteri G, Roggero PP, Garau G (2021) Evaluation of *Cynara cardunculus* L. and municipal solid waste compost for aided phytoremediation of multi potentially toxic element–contaminated soils. Environ Sci Pollut Res 28:3253–3265. 10.1007/s11356-020-10687-2 10.1007/s11356-020-10687-2 PMC7788029 32910403

[CR17] Gondek M, Weindorf DC, Thiel C, Kleinheinz G (2020) Soluble salts in compost and their effects on soil and plants: a review. Compost Sci Util 28:59–75. 10.1080/1065657X.2020.1772906

[CR18] Haney RL, Franzluebbers AJ, Jin VL, Johnson M, Haney EB, White MJ, Harmel RD (2012) Soil organic C: N vs. water extractable organic C:N. Open J Soil Sci 2:269–274

[CR19] Hu Y, Yu Z, Fang X, Zhang W, Liu J, Zhao F (2020) Influence of mining and vegetation restoration on soil properties in the eastern margin of the Qinghai-Tibet plateau. Int J Environ Res Public Health 17:4288. 10.3390/ijerph17124288 32560083 10.3390/ijerph17124288 PMC7344658

[CR20] Huang CL, Schulte EE (1985) Digestion of plant tissue for analysis by ICP emission spectroscopy. Commun Soil Sci Plant Anal 16:943–958. 10.1080/00103628509367657

[CR21] Ippolito JA, Barbarick KA, Brobst RB (2014) Copper and zinc speciation in a biosolids-amended, semiarid grassland soil. J Environ Qual 43:1576–1584. 10.2134/jeq2014.02.0074 25603243 10.2134/jeq2014.02.0074

[CR22] Ippolito J, Li L, Banet T, Brummer J, Buchanan C, Betts A, Scheckel K, Basta N, Brown S (2024) Soil health as a proxy for long-term reclamation success of metal-contaminated mine tailings using lime and biosolids. Soil Environ Health 2024:100096 10.1016/j.seh.2024.100096 PMC11534064 39498165

[CR23] Kabata-Pendias A (2011) Trace elements in soils and plants, 4th edn. CRC Press, Boca Raton

[CR24] Kok FS, Muhamad II, Lee CT, Razali F, Pa’e N, Shaharuddin S (2012) Effects of pH and temperature on the growth and β-glucosidase activity of Lactobacillus rhamnosus NRRL 442 in anaerobic fermentation. Int Rev Chem Eng 4(3):293–299

[CR25] Liebig MA, Miller ME, Varvel GE, Doran JW, Hanson JD (2004) AEPAT: Software for Assessing Agronomic and Environmental Performance of Management Practices in Long-Term Agroecosystem Experiments. Agron J 96:109–115

[CR26] Mehlich A (1984) Mehlich 3 soil test extractant: a modification of Mehlich 2 extractant. Commun Soil Sci Plant Anal 15:1409–1416

[CR27] Mukhopadhyay S, Maiti SK, Masto RE (2014) Development of mine soil quality index (MSQI) for evaluation of reclamation success: a chronosequence study. Ecol Eng 71:10–20

[CR28] Mukhopadhyay S, Masto RE, Yadav A, George J, Ram LC, Shukla SP (2016) Soil quality index for evaluation of reclaimed coal mine spoil. Sci Total Environ 542:540–550 26524272 10.1016/j.scitotenv.2015.10.035

[CR29] National Research Council (2005) Mineral tolerance of animals: second revised edition, 2005. The National Academies Press, Washington, DC. 10.17226/11309

[CR30] Ruiz F, Perlatti F, Oliveira DP, Ferreira TO (2020) Revealing Tropical Technosols as an Alternative for Mine Reclamation and Waste Management. Minerals 10:110. 10.3390/min10020110

[CR31] Shrestha P, Gautam R, Ashwath N (2019) Effects of agronomic treatments on functional diversity of soil microbial community and microbial activity in a revegetated coal mine spoil. Geoderma 338:40–47. 10.1016/j.geoderma.2018.11.038

[CR32] Sikdar A, Wang J, Hasanuzzaman M, Liu X, Feng S, Roy R, Sial TA, Lahori AH, Arockiam Jeyasundar PGS, Wang X (2020) Phytostabilization of Pb-Zn mine tailings with amorpha fruticosa aided by organic amendments and triple superphosphate. Mol Basel Switz 25:1617. 10.3390/molecules25071617 10.3390/molecules25071617 PMC7181007 32244753

[CR33] Spargo A, Doley D (2016) Selective coal mine overburden treatment with topsoil and compost to optimise pasture or native vegetation establishment. J Environ Manag 182:342–350. 10.1016/j.jenvman.2016.07.095 10.1016/j.jenvman.2016.07.095 27497311

[CR34] Sun X, Sun M, Chao Y, Shang X, Wang H, Pan H, Yang Q, Lou Y, Zhuge Y (2023) Effects of lead pollution on soil microbial community diversity and biomass and on invertase activity. Soil Ecol Lett 5:118–127. 10.1007/s42832-022-0134-6

[CR35] Trimarco T, Brummer JE, Buchanan C, Ippolito JA (2023) Tracking soil health changes in a management-intensive grazing agroecosystem. Soil Syst 7:94. 10.3390/soilsystems7040094

[CR36] Umeobi EC, Azuka CV, Ofem KI, Obite SU, Ezea CA, Abraham II, Alungbe ME, Akubue JC, John K, Ezeaku PI (2024) Evaluation of soil pollution effects on maize (Zea mays) at selected Pb–Zn and limestone mine sites in Ebonyi State, Southeastern Nigeria. Environ Monit Assess 196:768. 10.1007/s10661-024-12868-9 39080074 10.1007/s10661-024-12868-9

[CR37] Yost MA, Veum KS, Kitchen NR, Sawyer JE, Camberato JJ, Carter PR, Ferguson RB, Fernández FG, Franzen DW, Laboski CA, Nafziger ED (2018) Evaluation of the Haney Soil Health Tool for corn nitrogen recommendations across eight Midwest states. J Soil Water Conserv 73:587–592. 10.2489/jswc.73.5.587

